# Peer teaching medical students during a pandemic

**DOI:** 10.1080/10872981.2020.1772014

**Published:** 2020-06-04

**Authors:** Victoria Roberts, Katie Malone, Paul Moore, Tamarind Russell-Webster, Rachel Caulfield

**Affiliations:** Bristol Medical School, University of Bristol, Bristol, UK

**Keywords:** Peer to peer teaching, Covid-19, Peer teaching, medical student, undergraduate

## Abstract

Our personal views about the challenges of continuing to deliver peer teaching during a pandemic. We are a group of 4^th^ year medical students who are part of a student society which has delivered structured, highly formulaic peer-led teaching sessions for the past three years. During the COVID-19 pandemic, the reduced access to our normal clinical teaching highlighted the importance of peer-led teaching sessions. We wanted to continue with our peer-taught sessions but knew we would have to devise a new format to make our teaching accessible to our peers wherever they were. Here, we describe the challenges of online peer teaching during the COVID-19 pandemic and our reflections of the future implications to our group.

## Opinion

We have been running peer-led teaching sessions for the past three years as an affiliate society of our university medical society. Our teaching model has a system-based approach in which we run small group tutorials on different aspects of that week’s chosen system for a group of first year medical students. Teaching sessions are prepared and delivered by medical students in later years using PowerPoint. ‘Tutor’ signups often fill within minutes of release. The society also delivers a highly popular mock OSCE for the second year students and had plans to hold clinical skills sessions for the third year students. It was during the planning stage for these sessions that we received a series of announcements that brought this all to a halt. Firstly, our clinical placements were stopped, with no indication of when we would return [1]. This was followed by a university wide closure for a prolonged Easter break to allow the university to transition to online teaching in time for the beginning of the next semester. Finally, the UK government announced plans for a nationwide lockdown. As with the rest of the country, life as we knew it stopped.

With medical students at our university without teaching for at least 4 weeks whilst the university transitioned to its new way of teaching, our society received increasing requests from potential ‘learners’ and tutors alike to provide some kind of teaching. After conducting an informal survey of the students and with the premise of the sessions allowing an opportunity for revision for the tutors whilst delivering curriculum relevant content for the learners, we decided on two teaching branches. Fourth year students would teach third year students symptom-based medicine and surgery topics and third year students would teach second year students systems-based pathology. This was to be delivered through a video conferencing platform in a similar format to the live sessions.

The restructuring of the format of peer teaching was not without challenges. Studies have consistently found that the key practical challenges in delivering effective online teaching are inadequate technology skills among tutors and issues with the functionality and reliability of the online platform used [2]. As such we prioritised finding a suitably reliable and robust platform for the delivery of these new sessions and re-training tutors on how to deliver this new type of session. However, these paled in comparison to our main challenge of trying to maintain the personal aspect of peer teaching through a screen. In order to deliver an engaging tutorial, we believe that it should involve interaction between the tutor and the learners. As such, our society has always cultivated a more informal setting that allows the learners to freely ask questions without fear of judgement from their peers. However, with an online video chat, this atmosphere has become hard to replicate. There is a loss of non-verbal cues meaning that, from the tutors perspective, it is harder to establish the mood of the group. For example, if the group looks puzzled during the presentation, this is easily picked up on with face to face interaction and in response, the pace can then be altered or a concept re-explained without anyone having to say anything. From the learner’s perspective, as they too cannot see the reactions of their peers, they may feel more isolated and lose confidence in their ability to answer and ask questions.

Online teaching gives the learners the ability to be much more passive in the tutorial by not being as present. We have found this particularly to be the case with learners switching off their cameras and microphones during the sessions. Those students with their cameras switched off appeared less engaged, creating a challenging teaching environment for the ‘tutors’. Sessions that were normally lively and interactive turned into tutors talking to a blank screen, unsure if the students they are teaching were still on the call. However, we do not believe the learners lack a desire to be there as the sessions are optional, the tutors received good feedback and the learners have continued to sign up for more sessions. Interestingly, this passivity was seen in third year learners despite them complaining of being on the receiving end of it themselves when in the role of tutor for the second year students.

Aiming to find a solution to the situation, we sought to find if this behaviour had been observed previously. We found a study which compared face-to-face teaching with live teaching via an online platform among dental students [3]. This observed very similar outcomes to our online sessions with student-teacher interaction and student comfort significantly lower in the online sessions [3]. In fact, the students in this study preferred the idea of watching a recording of the online teaching session rather than participating in the original live version [3]. In response to this, we ensured that all of our sessions were recorded so that all students, regardless of whether they had attended the live online sessions or not, could review the sessions in their own time.

Despite the drawbacks, we saw the positive results of our new initiative very quickly. Between the two branches, we were teaching up to 25 sessions a week, with a mean audience of about 12 students per session (maximum capacity 15). The sign-up sheets were filled rapidly with students eager to both tutor and learn. Since it was announced that our exams would not contribute to our progression to the next year, the focus of the sessions has shifted more towards the practical application of the topic rather than model answers for exam-style questions. Whilst they still feature in the sessions, their lessening of importance has allowed the tutor to impart real life experience from the wards rather than just teach learners how to pass exams! Above all else, peer teaching has played its part in fostering a sense of community in what is a very isolating time. The regularity of the sessions restored some resemblance of structure to the lives of those involved.

Whilst we have been dispersed isolating across the world, we are united by being medical students at the same university. We acknowledge the strengths of online teaching, namely that it has given us the opportunity to continue teaching during this time but, in our opinion, on its own it is not equitable to our face to face sessions. We will reflect whether we could integrate it into our regular teaching in the future. However, when lockdown ends and life gradually goes back to normal, we will gladly return to our usual face to face teaching sessions.
